# Identification of prefoldin amplification (1q23.3-q24.1) in bladder cancer using comparative genomic hybridization (CGH) arrays of urinary DNA

**DOI:** 10.1186/1479-5876-11-182

**Published:** 2013-08-01

**Authors:** Virginia López, Pilar González-Peramato, Javier Suela, Alvaro Serrano, Ferrán Algaba, Juan C Cigudosa, August Vidal, Joaquim Bellmunt, Oscar Heredero, Marta Sánchez-Carbayo

**Affiliations:** 1 Tumor Markers Group, Molecular Pathology Program, Spanish National Cancer Center, Melchor Fernandez Almagro 3, Madrid E-28029, Spain; 2 Hospital Universitario La Paz, Pathology Department, Madrid, Spain; 3 Molecular Cytogenetics Group and Centre for Biomedical Research on Rare Diseases (CIBERER), Human Genetics Program, Spanish National Cancer Center, Madrid, Spain; 4 Urology Department, Hospital Universitario de Guadalajara, Guadalajara, Spain; 5 Fundació Puigvert, Pathology Department, Barcelona, Spain; 6 Hospital Universitari de Bellvitge, Pathology Department, L’Hospitalet de Llobregat, Barcelona, Spain; 7 Hospital del Mar, Oncology Department, Barcelona, Spain; 8 Urology Department, Hospital Universitario de Salamanca, Salamanca, Spain; 9 Proteomics Unit, CIC bioGUNE, Bizkaia Technology Park, Derio E-48160, Spain

**Keywords:** Bladder cancer, Array-CGH, FISH, Immunohistochemistry, Urine, Tissue arrays

## Abstract

**Background:**

Array-CGH represents a comprehensive tool to discover genomic disease alterations that could potentially be applied to body fluids. In this report, we aimed at applying array-CGH to urinary samples to characterize bladder cancer.

**Methods:**

Urinary DNA from bladder cancer patients and controls were hybridized on 44K oligonucleotide arrays. Validation analyses of identified regions and candidates included fluorescent in situ hybridization (FISH) and immunohistochemistry in an independent set of bladder tumors spotted on custom-made tissue arrays (n = 181).

**Results:**

Quality control of array-CGH provided high reproducibility in dilution experiments and when comparing reference pools. The most frequent genomic alterations (minimal recurrent regions) among bladder cancer urinary specimens included gains at 1q and 5p, and losses at 10p and 11p. Supervised hierarchical clustering identified the gain at 1q23.3-q24.1 significantly correlated to stage (p = 0.011), and grade (p = 0.002). The amplification and overexpression of Prefoldin (PFND2), a selected candidate mapping to 1q23.3-q24.1, correlated to increasing stage and tumor grade by means of custom-designed and optimized FISH (p = 0.013 and p = 0.023, respectively), and immunohistochemistry (p ≤0.0005 and p = 0.011, respectively), in an independent set of bladder tumors included in tissue arrays. Moreover, PFND2 overexpression was significantly associated with poor disease-specific survival (p ≤0.0005). PFND2 was amplified and overexpressed in bladder tumors belonging to patients providing urinary specimens where 1q23.3q24.1 amplification was detected by array-CGH.

**Conclusions:**

Genomic profiles of urinary DNA mirrowed bladder tumors. Molecular profiling of urinary DNA using array-CGH contributed to further characterize genomic alterations involved in bladder cancer progression. PFND2 was identified as a tumor stratification and clinical outcome prognostic biomarker for bladder cancer patients.

## Introduction

Bladder cancer represents the 4th most common malignancy among men and the 8th cause of male cancer deaths. Approximately 90% of malignant tumors arising in the uroepithelium of the bladder are transitional cell carcinomas (TCC) [1]. Currently, the diagnosis and surveillance of bladder cancer is based on the information provided by cystoscopy, considered the gold standard, in combination with urinary cytology findings. The invasive nature of cystoscopy, still uncomfortable for a great number of individuals, together with the subjective nature of urinary cytology, greatly dependant on the skills of the pathologist and the quality of the sample [2], triggers for the search of non-invasive objective methods for bladder cancer. Urinary specimens, in direct contact with bladder tumors, represent easily attainable samples to explore molecular events associated with tumor progression and provide biomarkers for cancer detection, surveillance and clinical outcome stratification.

Gains and losses of DNA copy numbers in specific chromosomal regions are frequent and critical genomic changes associated with tumor development and progression. These genomic imbalances can be detected by conventional CGH on metaphase spreads [3], or DNA sequences spotted into an array (array-CGH) [4]. In bladder cancer, conventional CGH was applied in frozen TCCs alone [5-9], or including squistosoma-associated squamous cases [10]. Analyses were also performed using paraffin-embedded TCCs alone [11-13], or including squamous tumors [10,14,15], and cell lines [16-18]. Array-CGH, using Bacteria Artificial Chromosome (BAC) or oligonucleotide arrays, represents a sensitive method for high-resolution analysis of genomic imbalances, able to detect small amplicons and deletions. In bladder cancer, BAC arrays were applied in frozen bladder tumors [19-24], and cell lines [25,26]. Oligonucleotide-based array-CGH was utilized to define the DNA copy number changes even in paraffin-embedded tumors [27]. These reports showed that array-CGH was widely explored in bladder tumor specimens and cell lines. A recent report has applied a focused miniarray-CGH test to body fluids [28]. In this study, we aimed at evaluating high- throughput array-CGH profiling of urinary DNA of bladder cancer patients as a means contributing to further characterize genomic alterations involved in tumor progression and identify potential bladder cancer biomarkers (Additional file 1: Figure S1).

## Methods

### Urinary DNA specimens

Urinary samples belonging to patients with primary bladder tumors (n = 14) were obtained following institutional reviewed approved protocols at participating institutions. Samples were collected in compliance with the Helsinki Declaration, after written informed consent according to SAF2009-13035 and SAF2012-40206 ethical approvals. The presence of the disease was confirmed by cystoscopy, together with the histopathologic information after surgical interventions. Urinary specimens positive for bladder cancer were obtained from 5 pTa, 4 pT1, 4 pT2 and 1 pT4 cases (Table 1). Genomic DNA from urine samples belonging to healthy donors and individuals with no evidence of disease, were used to generate two normal DNA reference pools (n = 8) (Table 1). Urinary DNA was extracted using the QIAamp DNA Micro kit (Qiagen, Hilden, Germany). Concentration and purity of DNA samples were determined with a ND-1000 spectrophotometer (NanoDrop Technologies, Wilmington, DE).

**Table 1:** Demographic and histopathologic information of the bladder cancer patients and controls providing urinary samples utilized for DNA profiling using oligonucleotide array-CGH

**Bladder Cancer Patients**				
**ID**	**Gender **^**a**^	**Age**	**Stage**	**Grade**
105 HG	M	65	**pTA**	G1
125 HG	F	51	**pTA**	G1
130 HG	M	54	**pTA**	G1
136 HG	M	58	**pTA**	G1
138 HG	F	70	**pTA**	G3
75 HS	M	75	**pT1**	G2
123 HG	M	78	**pT1**	G2
131 HG	M	81	**pT1**	G2
141 HG	M	72	**pT1**	G3
127 HG	M	79	**pT2**	G3
132 HG	M	74	**pT2**	G3
139 HG	M	69	**pT2**	G3
129 HG	M	66	**pT2**	G3
100 HG	M	78	**pT4**	G3
**Controls**				
**ID**	**Gender **^**a**^	**Age**	**Pool**	**Status**
DNA 19/10	F	30	1	Healthy donor
76HS	M	83	1	No evidence of disease
79HS	F	73	1 & 2	Cystitis
111HG	M	55	1	No evidence of disease
116HG	F	61	2	Healthy donor
118HG	M	61	1 & 2	No evidence of disease
135HG	M	51	2	Healthy donor
143HG	F	71	2	Healthy donor

^a^*M*, Male; *F*, Female.

### Urinary DNA labelling and hybridization to the Array-CGH platform

Genomic DNA (250 ng) from urinary specimens (n = 22) belonging to bladder cancer patients, healthy donors and individuals with no evidence of disease (reference pools) were hybridized against Human Genome CGH 44 k oligonucleotide microarrays version B, (Agilent, Palo Alto, CA). This array consisted of 44000 (60-mer) oligonucleotide probes, 40,912 of them against known genes and Expressed Sequenced Tag (ESTs), at a mean resolution of 75 Kb. Urinary DNA from bladder cancer patients was labelled with Cyanine 5 (Cy5) and urinary DNA reference pool with Cyanine 3 (Cy3). Arrays were scanned at 670 and 570 nm for Cy5 and Cy3 respectively, using the G2565BA Scanner (Agilent).

### Array-CGH imaging and data analysis

Microarray images were transformed to fluorescence intensities using the Feature Extraction Software, v9.5 (Agilent), allowing spot gridding, quantification and local background subtraction. Microarray data were visualized using the CGH Analytics v3.3 software (Agilent). The hybridization of the urinary samples was assessed according to the quality control parameter Derivative Log Ratio (DLR) spread provided by this software. The DLR spread metrics estimated the spread of log ratio differences between consecutive probes along all chromosomes. Samples were proven to exhibit a DLR spread lower than 0.3 log units and a signal to noise ratio for each channel greater than 30.

Chromosome segmentation was carried out using the smoothing algorithm from the InSilicoArray CGH software at http://www.gepas.org (GEPAS, Valencia, Spain) [29], which estimates the mean log_10_ ratio value of all the probes belonging to a given chromosomal region, and provides the Copy Number Value (CNV) of such region. These CNVs were checked under the threshold of +0.1 for gains and −0.1 for losses allowing the identification of the chromosomal aberrations in the urinary specimens under study. Amplifications were defined if consecutive probes spanning a gained region, showed a CNV higher than 0.3979 (more than five DNA copies) [29]. Finally, the CNVs were categorized as 0, 1 or −1 (indicating no change, gain or loss, respectively) in order to identify minimal recurrent regions with overlapping gains or losses affecting different urinary specimens. A region was considered to harbour a minimal recurrent region if at least three consecutive probes were simultaneously changed in at least two specimens. Single-probe aberrations were not scored as copy number changes. DNA copy changes observed in the samples showing at least 80% of their sequence overlapping with known polymorphisms included in the Database of Genomic Variants were excluded from the analyses (http://projects.tcag.ca/variation).

### Supervised hierarchical clustering

In order to evaluate the association of copy number changes detected by array-CGH and clinicopathologic variables, the minimal recurrent regions of gains and losses were subjected to supervised hierarchical clustering using the POMELO tool from the ASTERIAS software (http://www.asterias.bioinfo.cnio.es). Obtained p-values using the analysis of variance (ANOVA) test were adjusted for false discovery rates (FDR) corrections [30].

### Tissue microarrays

We constructed different tissue microarrays including triplicate cores of the paired bladder tumors belonging to the patients providing urinary specimens and independent sets of primary TCCs cases (n = 181) with available follow-up, recruited from several collaborating clinical institutions. Samples were collected in compliance with the Helsinki Declaration, after written informed consent according to SAF2009-13035 and SAF2012-40206 ethical approvals. For tissue array construction, tumor tissues were embedded in paraffin and five-μm sections were stained with hematoxylin and eosin to identify viable, morphologically representative areas of the specimen from which needle core samples were taken, using a precision tissue microarrayer (Beecher Instruments, Silver Spring, MD). From each specimen, triplicate or quadruplicate cores with diameters of 1.0 mm were punched and arrayed on the recipient paraffin block. The distribution of tumor stage among the bladder tumors spotted onto the tissue arrays was: pT1 (78), pT2 (59), pT3 (26), pT4 (18), while their tumor grade was: grade 2 (10), and grade 3 (171). Patients were treated surgically by transurethral resection in non-invasive lesions and cystectomy in muscle-invasive tumors. Adjuvant therapy consisted of intravesical instillations with the Bacille of Calmette-Guerin in non-muscle invasive disease and cisplatin chemotherapy in muscle-invasive tumors, respectively.

### Fluorescence-*in-situ*-hybridization analyses (FISH)

FISH analysis was performed on the tissue arrays mentioned above. Four BACs covering the 1q23.3 region where *PFND2* maps: RP11-157H6, RP11-297K8, RP11-1008K23, and RP11-136J10, were selected from UCSC (http://genome-ucsc.edu.) and labelled using the Spectrum Red 2’-deoxyuridine 5’-triphosphate (dUTP) (Vysis, Downers Grove, IL) by nick translation with the CGH Nick Translation kit (Vysis). Four independent BACs mapping to 1p were selected as controls: RP11-199O1, RP11-624A15, RP11-709H9, and RP11-473K14, and were labelled with the Spectrum Green dUTP (Vysis). BACs were obtained from the BACPAC Resource Center (Oakland, CA, US). High hybridization efficiency and specificity were confirmed by performing FISH on normal lymphocyte metaphase preparations.

FISH evaluation was performed using a fluorescence microscope (Olympus BX61, Olympus Corporation, Tokyo, Japan). Images were captured and analysed using the Cytovision image analysis system (Applied Imaging Ltd., New Castle, UK). Only discrete signals in nuclei with distinct nuclear border stained with 4’ , 6-diamidino-2-phenylindole (DAPI) were counted. Overlapping nuclei were excluded from evaluation. Five different categories were defined for classification and interpretation of FISH results. The observation of two red and two green signals per nucleus was considered ‘normal’ and categorized as ‘1’; such pattern combined with three red spots in a similar percentage was categorized as ‘2’; copy number ‘gains’ were defined if three red spots were present in at least 50 % of intact tumor nuclei, being categorized as ‘3’. Category ‘4’ was defined if there were similar percentages of nuclei showing three than those with more than three red signals. Finally, if at least 50% of the tumor cells doubled the red to green spots ratio, the case was considered ‘amplified’ and categorized as ‘5’.

### Immunohistochemistry (IHC)

Protein expression patterns of PFND2 were assessed by IHC analysis on the tissue arrays mentioned above (n = 181), using avidin-biotin immunoperoxidase procedures [31]. A goat polyclonal antibody against PFND2 from IMGENEX (San Diego, CA) was applied at a 1:800 dilution. Ki67 was assessed using a mouse monoclonal antibody diluted at 1:100 (clone MIB-1; DAKO, Glostrup, Denmark). The absence of the primary antibody was used as negative control. Diaminobenzidine was the final chromogen and hematoxylin was the nuclear counterstain.

### Cell lines and western blotting analysis

Nine bladder cancer cell lines derived from TCCs of the bladder were obtained from the American Type Culture Collection (Rockville, MD, US), grown, and collected under standard tissue culture protocols. The specificity of the antibody utilized for IHC mentioned above was screened by Western blotting at 1:300 dilution using 75 μg of lysate protein per lane. An alpha-tubulin antibody (mouse monoclonal, 1:5000 dilution, Sigma- Aldrich, St. Louis) was utilized as loading control.

### Statistical methods

The association between gene and protein expression measured on tissue arrays by FISH and IHC respectively, and histopathologic stage and tumor grade was evaluated using the non-parametric Wilcoxon-Mann–Whitney and Kruskall-Wallis tests [32]. Associations with disease-specific overall survival were estimated using the log-rank test in those cases for which follow-up information was available. Disease-specific overall survival time was defined as the years elapsed between transurethral resection or cystectomy and death as a result of disease (or the last follow-up date). Patients who were alive at the last follow-up or those lost to follow-up were censored. There is no consensus on the cutoffs for the immunohistochemical expression for protein expression patterns for prefoldin. The number of cells expressing a cytoplasmic sublocalization was analyzed continuously. The cutoff value for low, medium and high expressing cases was specified at the median percentage score of positive cytoplasmic tumor cells resulting in a value of intensity of 2 (++). Prefoldin intensity was then analyzed taking the cutoff of 2 (++) when considered as a categoric variable. Survival curves were plotted using the Kaplan-Meier methodology [32]. Statistical analyses were performed using the SPSS statistical package v17.0 (SPSS Inc., Chicago, IL).

## Results

Reproducibility Assessment of Array-CGH using Urinary DNA. Owing to the limited amount of DNA extracted from urinary specimens, the minimum quantity of initial urinary DNA necessary to obtain reliable CGH estimations was initially tested. Reverse labelling (dye swaps) analyses were performed comparing 250 *versus* 500 ng as the starting amount of genomic DNA. Gains and losses using 500 ng were also detected using 250 ng (Additional file 2: Figure S2A). The optimal correlation found between 250 *versus* 500 ng revealed that the use of the lowest DNA amount (250 ng) did not prevent to detect any relevant copy number changes, providing reliable genomic profiling array-CGH.

The potential influence of varying the source of non-neoplastic DNA in the reference pool from several donors was tested. Two urinary pools including different healthy donors and individuals with no evidence of disease (confirmed by cystoscopy), were compared by array-CGH (Additional file 2: Figure S2B). The lack of significant copy number changes among these pools when performing reverse labelling hybridizations revealed that variations in the source of non-neoplastic DNA in the reference pool did not prevent detecting relevant copy number changes of bladder cancer patients. Overall, these analyses revealed that array-CGH was feasible in bladder cancer urinary specimens using a low initial amount of DNA. Additionally, variations in the source of healthy normal urothelium in the reference pool did not impact on the identification of bladder cancer associated genomic changes.

### Identification of genomic imbalances in urinary specimens

**Figure 1:**
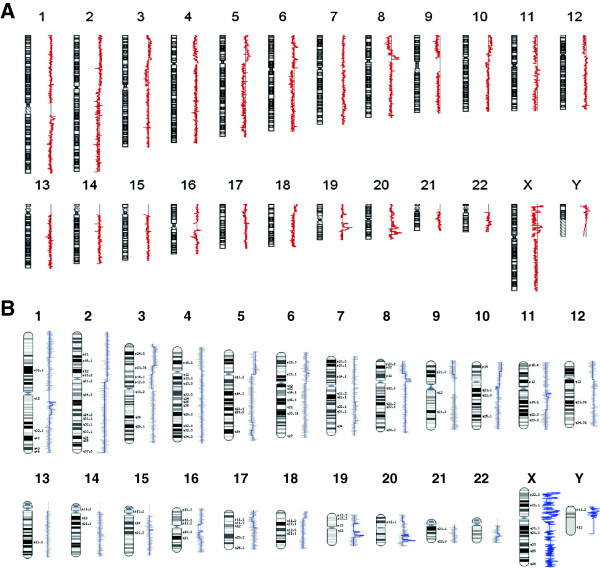
**Array-CGH detected genomic imbalances in urinary specimens. A**. Summary ideogram given by the CGH Analytics software for a representative urinary belonging to a patient with a pT1G2 bladder tumor (case 131HG). Average log_2_ ratio values along the chromosomes are represented by the red line. Displacement of the tracing of this red line to the right or left represents genomic gains or losses, respectively. The ideograms are ordered from chromosome 1 to 22, including chromosomes X and Y as well **B**. Detailed genomic DNA profile image for the same case obtained using the InSilicoArray CGH software. Average log_10_ ratio values of the CNVs along the chromosome are represented by the blue line. Displacement of the tracing of this blue line to the right or left represents genomic gains or losses, respectively. The profiles are ordered from chromosome 1 to 22, including chromosomes X and Y as well.

The high-resolution genomic analysis for the urinary specimens belonging to bladder cancer patients was initially assessed using the CGH Analytics (Figure 1A), and the InSilicoArray CGH softwares (Figure 1B). Additional file 3: Figure S3 provides visualization for all of them.

Examination of CNVs served to identify chromosomal regions of gain and loss among the urinary specimens (Table 2). The group of urinary specimens belonging to patients with papillary pTa lesions did not show gains and harboured 30% of the losses. Non muscle-invasive pT1 cases displayed the highest number of copy number changes, showing 86.96% of the gains (20/23), and 70% of the losses (7/10). Muscle-invasive pT2+ cases displayed 17.39% of the gains (4/23), and 10% of the losses (1/10). Regarding tumor grade, urines belonging to patients with grade 1 tumors did not show genomic gains, and harboured 30% of the losses (3/10). Grade 2 cases displayed the highest number of CNVs, showing 86.96% of the gains (20/23), and 70% of the losses (7/10). Urines belonging to grade 3 cases had 17.39% of the 23 gains (4/23), and 20%of the losses (2/10).

**Table 2:** Gains and losses detected by array-CGH (ordered by the CNVs) in each case

**CHROMOSOMAL REGIONS OF GAINS**							
**1st Probe Name**	**Chromosome band (Ensembl)**	**Start position**	**CNV**	**Cases**	**Stage**	**Grade**	**Number of probes**
A_14_P115961	19q13.12-q13.2	Chr19:041377289	0.41069	123HG	pT1	G2	152
A_14_P108613	20q13.13-20q13.2	Chr20:048671089	0.3998	123HG	pT1	G2	52
A_14_P123794	2p23.3	Chr2:027502237	0.31715	139HG	pT2	G3	5
A_14_P201430	20q12-q13.12	Chr20:039082984	0.30745	123HG	pT1	G2	101
A_14_P130062	19q12	Chr19:034522544	0.28517	123HG	pT1	G2	18
A_14_P118037	15q25.1	Chr15:077932195	0.27764	139HG	pT2	G3	8
A_14_P110668	8p12-p11.21	Chr8:036027406	0.26755	123HG	pT1	G2	120
A_14_P200557	19q13.42	Chr19:060805118	0.21635	139HG	pT2	G3	5
A_14_P128880	19p13.11	Chr19:017431158	0.20428	123HG	pT1	G2	68
A_14_P107769	15q21.2	Chr15:048417834	0.19587	123HG	pT1	G2	13
A_14_P119367	17q12	Chr17:034473380	0.17478	123HG	pT1	G2	32
A_14_P114294	11q12.3	Chr11:061766567	0.16714	123HG	pT1	G2	44
A_14_P108406	10p15.3-p12.31	Chr10:001070037	0.16666	123HG	pT1	G2	217
**A_14_P103528**	**1q21.2-q21.3**	**Chr1: 146938814**	**0.16485**	**123HG**	**pT1**	**G2**	**141**
				**131HG**	**pT1**	**G2**	
**A_14_P126330**	**1q24.2-q24.3**	**Chr1:163305648**	**0.13374**	**123HG**	**pT1**	**G2**	**122**
				**131HG**	**pT1**	**G2**	
**A_14_P126727**	**1q23.3-q24.1**	**Chr1:157564589**	**0.12808**	**75HS**	**pT1**	**G2**	**116**
				**123HG**	**pT1**	**G2**	
				**131HG**	**pT1**	**G2**	
A_14_P102488	6p21.1	Chr6:041358388	0.12681	123HG	pT1	G2	83
A_14_P135779	18p11.32-p11.21	Chr18:000170229	0.12203	123HG	pT1	G2	176
A_14_P105338	3p26.1-p21.33	Chr3:006615679	0.12067	123HG	pT1	G2	529
**A_14_P101810**	**5p13.33-p12**	**Chr5:000148243**	**0.11948**	**123HG**	**pT1**	**G2**	**440**
				**132HG**	**pT2**	**G3**	
A_14_P200670	16q11.2-q12.1	Chr16:045172598	0.11639	123HG	pT1	G2	49
A_14_P105981	22q12.2-q13.1	Chr22:030417113	0.10517	123HG	pT1	G2	162
A_14_P126618	7q21.2-q33	Chr7:091892016	0.10403	123HG	pT1	G2	631
**CHROMOSOMAL REGIONS OF LOSSES**							
**1st Probe Name**	**Chromosome band (Ensembl)**	**Start position**	**CNV**	**Cases**	**Stage**	**Grade**	**Number of probes**
A_14_ P135773	13q14.2-q14.3	Chr13:047555252	−0.26486	132HG	pT2	G3	66
**A_14_ P139280**	**11p15.5**	**Chr11:000283643**	**−0.18187**	**130HG**	**pTA**	**G1**	**6**
				**75HS**	**pT1**	**G2**	
**A_14_ P134493**	**10p15.3**	**Chr10:000138206**	**−0.16050**	**136HG**	**pTA**	**G1**	**9**
				**138HG**	**pTA**	**G3**	
A_14_ P110624	9p24.3-p21.2	Chr9:000204367	−0.15219	123HG	pT1	G2	324
A_14_ P128129	10q11.22-q21.1	Chr10:047954413	−0.12787	123HG	pT1	G2	65
A_14_ P110069	2q37.1-q37.3	Chr2:233099731	−0.12386	131HG	pT1	G2	167
A_14_ P119514	8p23.3-p21.2	Chr8:000181530	−0.12229	123HG	pT1	G2	309
A_14_ P109355	16p11.2-p11.1	Chr16:031804884	−0.11781	123HG	pT1	G2	11
A_14_ P112424	7q22.1	Chr7::099453161	−0.11741	130HG	pTA	G1	75
A_14_ P103261	5q33.3-q35.1	Chr5:159767536	−0.11684	123HG	pT1	G2	95

The minimal recurrent regions of gains and losses are highlighted in bold.

The CNVs for the regions of gain and loss were revised to identify overlapping (minimal recurrent) gained and lost regions among the urinary specimens, highlighted in Table 2. The most recurrent genomic alteration was the gain at 1q23.3-q24.1, followed by gains at 1q21.2-q21.3, 1q24.2-q24.3 and 5p13.33-p12, and losses at 10p15.3 and 11p15.5. The complete set of known genes mapping to these minimally recurrent regions is provided as Additional file 4: Table S1. Overall, these analyses revealed the utility of array-CGH as a high-throughput technique to identify genomic changes associated with bladder cancer using urinary specimens. Additionally, they revealed that the gain at 1q21-q24 was of potential clinical interest for urinary biomarkers discovery.

### Supervised hierarchical clustering identified 1q23.3-q24.1 differentially expressed region regarding histopathologic variables

**Figure 2:**
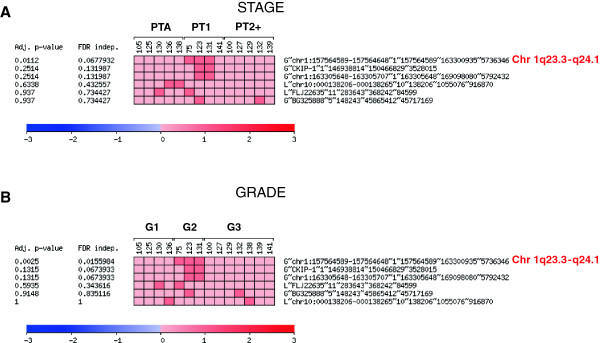
**Supervised clustering of the minimal recurrent regions revealed the association of the gain at 1q23.3- q24.1 with histopathologic variables.** The genomic profiles of the urinary specimens belonging to bladder cancer patients were clustered using the POMELO software based on: **A**. tumor stage, and **B**. tumor grade. The figures illustrate the association of the minimal recurrent regions of gain (G) and loss (L), with these histopathologic variables including the information of the first gene mapping at each region and their respective p-values and FDRs.

**Figure 3:**
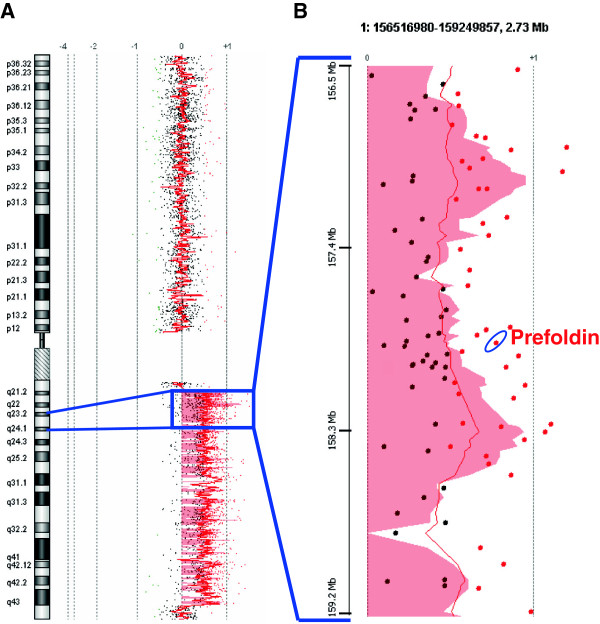
**Array-CGH analysis showing the 1q23.3- q24.1 region that harbors the PFND2 gene. A**. The ideogram of chromosome 1 for the urinary specimen **131HG** belonging to a **pT1G2** bladder tumor is shown to the left. The central red tracings represent the mean signal ratio of each of the clones along the chromosome generated by the CGH Analytics software. Displacement of this red line to the right of the centre indicates relative genomic gains. **B**. Gene view of the gain at 1q23.3-q24.1 displaying probes as dots. The color of each dot represents normal (black) or gains (red). The gain of **PFND2** identified by array-CGH is highlighted.

Supervised hierarchical clustering was performed to identify top discriminatory genomic imbalances among the minimal recurrent regions of gain and loss associated with tumor stage and grade, by means of ANOVA test analyses. Regarding tumor stage, 1q23.3-q24.1 was found differentially expressed between the urinary specimens (p = 0.011, FDR =0.068) (Figure 2A). 1q23.3-q24.1 was also the region differentially expressed regarding their tumor grade (p = 0.002, FDR = 0.016) (Figure 2B). Overall, these results confirmed the clinical relevance of the gain at 1q23.3-q24.1 as a minimal recurrent region associated with clinicopathologic variables of bladder cancer (Figure 3A). Localization of the minimal recurrent regions of aberration in the urinary specimens associated with histopathologic variables prompted us the search for genes potentially involved in bladder cancer in the most recurrent region at 1q23.3-q24.1. This region harboured a set of genes commonly gained in 3 out of the 14 urinary samples analyzed, with 116 probes showing this gain (Table 2). Among these genes identified in this region showing the highest log-ratio gains (Additional file 4: Table S1), PFND2 was selected for further validation analyses (Figure 3B).

### PFND2 is associated with tumor progression and clinical outcome of bladder cancer patients

**Figure 4:**
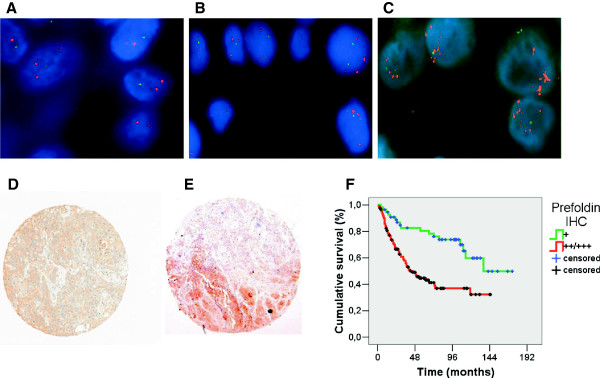
**PFND2 expression patterns are associated with tumor progression and clinical outcome. A**, **B**, **C**. Representative FISH images of **PFND2** on tissue arrays containing bladder tumors (n = 181) showing cases with normal **(A)**, gained **(B)**, and amplified **(C)** hybridization patterns. **D**.**E**. Representative immunostainings by immunohistochemistry showing cases with low (+) **(D)** and high (+++) **(E)** intensity of **PFND2** cytoplasmic expression on tissue arrays. **F**. Kaplan-Mayer curve survival analysis indicating that a cytoplasmic protein expression of **PFND2** with medium (++) or high (+++) intensity measured by immunohistochemistry on tissue arrays was associated with shorter disease-specific overall survival (log rank, p ≤0.0005).

The gain of PFND2 detected by array-CGH, and its protein overexpression were initially evaluated by FISH and IHC analysis, respectively, on the paired bladder tumors belonging to the bladder cancer patients providing urinary specimens. The cases showing PFND2 amplification in the urinary specimen displayed amplification in the paired bladder tumor by FISH (Additional file 1: Figure S1B) and also protein overexpression by IHC (Additional file 1: Figure S1C). Paired normal urothelium specimens showed a normal pattern of hybridization (data not shown, similar to Figure 4A). The association between FISH and IHC observations with clinicopathologic variables was then evaluated on tissue arrays containing independent sets of bladder tumors (n = 181). For FISH analyses, three main hybridization patterns were observed: normal (Figure 4A), gains (Figure 4B), and amplifications (Figure 4C). For IHC analyses, the intensity of PFND2 immunostaining was categorized from 1 (Figure 4D), to 3 (Figure 4E). Interestingly, tumor stage was significantly associated with the gene amplification observed by FISH (p = 0.013), and the protein overexpression of PFND2 observed by IHC (p ≤ 0.0005). Tumor grade was also associated with PFND2 amplification (p = 0.023), and its overexpression (p = 0.011). PFND2 amplification and protein overexpression were significantly associated between them (Kendall’s tau = 0.125, p = 0.034), and with increased proliferation as measured by Ki67 staining (Kendall’s tau τ = 0.223, p ≤ 0.0005, and τ = 0.433, p ≤ 0.0005, respectively). Furthermore, patients with high cytoplasmic PFND2 overexpression had shorter disease-specific survival than those with low expression (log rank, p < 0.0005, Figure 4F). Overall, FISH and IHC validation analyses on tissue arrays containing an independent large set of bladder tumors served to associate PFND2 amplification and overexpression with histopathologic variables of tumor progression and clinical outcome of bladder cancer patients.

## Discussion

Since its introduction, array-CGH served to discover underlying molecular mechanisms leading to tumorigenesis and cancer progression and the identification of potential biomarkers and therapeutic target candidates. The novelty of this study deals with the application of genome-wide copy number analysis to urinary specimens. Interestingly, array-CGH served to detect genomic alterations specific of bladder cancer in non-invasive specimens, including several previously reported alterations of known biological and clinical relevance in bladder tumors, and the precise refinement of the localization of copy number changes. Such genomic alterations were proven to be specific of bladder cancer cells on matching bladder tumors of the urinary specimens, and on independent sets of bladder tumors by means of FISH and IHC analyses. Thus, the present study showed that array-CGH on urinary specimens could mirrow bladder tumors and provided further information contributing to characterize bladder cancer progression and identifying candidate biomarkers for bladder cancer.

Several technical issues in our experimental design were critical to allow detecting relevant genomic changes in urinary specimens. First, the high size of the array (44000 probes) in combination with its resolution (75 Kb) allowed a refined screening of genomic imbalances even with the use of a low amount of starting genomic DNA (250 ng). Second, the chosen pool of urinary DNA including normal healthy donors and individuals without evidence of disease was appropriate enough to detect genomic changes characteristic of bladder tumors, regardless the variation of the control individuals providing urinary specimens. The choice of the reference DNA extracted from uroepithelial cells of healthy individuals or patients with benign conditions without bladder cancer is justified since this source of non-neoplastic uroepithelial cells are exposed to the urinary environment, similarly to the DNA obtained from the bladder cancer patients. The pool served to exclude non bladder cancer specific genomic changes due to normal cells present in the urine allowing the use of a conservative threshold for CNVs.

The high-resolution array-CGH analyses identified genomic gains and losses between urinary DNA belonging to bladder cancer patients and controls, many of which were previously reported using conventional CGH and array-CGH analyses on bladder tumor samples and cell lines. Finding genomic changes previously reported in bladder tumors provided confidence to our working hypothesis by which urinary specimens belonging to bladder cancer patients may mirrow genomic alterations present in bladder tumors. The gains we identified within minimal recurrent regions at 1q and 5p; and the amplifications that we found in these urinary specimens at 19q and 20q, the minimally recurrent regions of loss at 11p and 10p; and those showing the highest loss CNVs at 13q and 9p, were previously reported in bladder cancer cells and tumors [5-8,10-16,18,19,21,23-41]. Our study allowed the novel refinement of the localization of copy number changes in several regions including the gain at 19p13.11 or the loss at 10p15.3 among the most differentially changed and minimally recurrent regions. In a few urinary specimens, especially in cases with muscle-invasive tumors, small chromosome regions, previously reported to be altered in bladder cancer were not detected using the oligonucleotide array-CGH platform. In addition to the different type of bladder tumor analyzed in such studies (stage and differentiation patterns), this observation could be associated with a lower proportion of tumor cells as compared to the DNA corresponding to non-neoplastic epithelial cells in the urine of bladder cancer patients, which could be considered a potential limitation. Another explanation could be related to the potential presence of multiple clones in the urinary specimens [42], which could dilute the detection of cancer cells with genomic alterations, so that regions with few aberrant cancer cells could not be scored as copy number changes because of our conservative applied cutt-off [43]. In the urinary specimens analyzed, higher numbers of genomic alterations were observed in non-invasive pT1 lesions as compared to invasive tumors. The lower number of genomic alterations observed in muscle-invasive tumors could be related to the common solid pattern of growth of advanced tumors towards the inner muscular layer not allowing a high proportion of cancer cells to reach the urine as compared to the frequent papillary growth pattern towards the urine in non-invasive lesions.

In the present study, the most frequent genomic alterations detected in the urinary DNA were at the long arm of chromosome 1, with three different minimal recurrent regions of gain mapping at 1q21.2-q21.3, 1q23.3-q24.1, and 1q24.2-q24.3. Chromosome 1q gain was frequently reported by conventional CGH in bladder tumors: frozen TCC [5,6,8,36,38], paraffin-embedded TCC [11-13], and cell lines [16,18]. More recently, the application of array-CGH platforms allowed a precise mapping of the 1q gained regions in TCCs [19,21], adenocarcinomas [27], and cell lines [25]. Gains at 1q21.2-q21.3 were detected in muscle-invasive and squamous cells [25]. The 1q23.2-q24.1 region was found gained in muscle-invasive cells [25], and amplified in pT1 tumors [11,12]. At the 1q24.2-q24.3 region, gains were detected in TCCs [19], being such amplification at this region described in pT1 [11,12,21], and muscle-invasive bladder tumors [21]. The simultaneous gains and amplifications at these three regions at chromosome 1q were also described in TCC tumors [36], in schistosoma-associated TCCs [14], in TCC and SCC regardless of its association with squistosoma infections [10]; and even in rare small cell carcinomas of the bladder by conventional CGH [39]. Similarly to our results in urinary specimens, gains at 1q21-q24 were reported in pT1 but not in pTa TCCs [11,13,36], an observation suggesting that this region may carry candidate genes involved in progression into muscle-invasive disease. The novelty of our report deals with the precise refinement of gains at this chromosomal region using oligonucleotide arrays together with the identification of such alterations in urinary specimens.

Localization of minimal recurrent regions of aberration in the urinary samples prompted us the search for genes potentially involved in bladder cancer progression. Losses were most often observed in early stages (pTa), whereas chromosomal gains became the most frequent type of aberration in more aggressive pT1 tumors, as previously reported [36]. Although bladder cancer subclassification was not the main objective of our study because of the limited number of specimens analyzed, the supervised hierarchical clustering of the minimal recurrent regions highlighted 1q23.3-q24.1 as a relevant gained region in the urinary DNA belonging to bladder cancer patients regarding their histopathologic tumor stage and grade. PFND2 was selected as one of the top-ranked genes mapping at this region for which probes and antibody were available for further validation. Although this chromosome arm was previously described to be altered in bladder cancer by conventional CGH and array-CGH, as summarized above, to our knowledge, PDND2 had not been reported to be differentially expressed in bladder cancer. PFND2 is a chaperone heterooligomer protein involved in the folding of its target proteins [44,45]. This is relevant in cancer research since members of the actin-related protein family requiring the PFND2 pathway for their proper maturation play a role in a variety of key cellular events, including orientation of the spindle during mitosis, nuclear migration [46], membrane polarity and endocytosis [47], or transcriptional regulation [48,49]. The amplification and overexpression results presented in this report correlating between them and with increased proliferation rates are consistent with the upregulation of members of the PFND2 protein family in cells and tumors of different origin such as glioblastoma [50], breast [51], pancreatic [52], or colon [53], where they are believed to play an oncogenic role [54-56]. With this biological relevant information, PFND2 was selected because of availability of reagents to be optimized for its assessment in bladder tumors. Our study showed that urinary DNA reflected the alterations present in the tumor since validation analyses confirmed PFND2 to be amplified (Additional file 1: Figure S1B) and overexpressed (Additional file 1: Figure S1C) on paired bladder tumors of such urinary specimens. Consistent with the identification of PFND2 as one of the genes belonging to a region differentially gained regarding tumor stage and grade, the DNA and protein expression patterns of PFND2 were significantly associated with increasing tumor stage and grade in an independent large set of bladder tumors spotted on tissue arrays by two independent analytical methods at the DNA and protein level. Further research is warranted to dissect its biological role in bladder cancer using appropriate *in vitro* and *in vivo* models.

## Conclusions

PFND2 was identified as a candidate biomarker in bladder cancer. Importantly, PFND2 overexpression in uroepithelial malignancies suggested its potential utility as a tumor stratification and clinical outcome prognostic biomarker. Furthermore, its detection in urinary specimens suggests the potential of the measurement of this gene by FISH as a complementary adjunct of urinary cytology or as a protein biomarker for the diagnosis and follow-up of patients affected by uroepithelial malignancies.

## Abbreviations

ANOVA: Analysis of variance; BAC: Bacteria artificial chromosome; CGH: Comparative genomic hybridization; CNV: Copy number value; Cy5: Cyanine 5; DAPI: 4’ 6-Diamidino-2-phenylindole; DLR: Derivative log ratio; dUTP: 2’-Deoxyuridine 5’-triphosphate; EST: Expressed sequenced tag; FDR: False discovery rates; FISH: Fluorescence-*in-situ*-hybridization; IHC: Immunohistochemistry; TCC: Transitional cell carcinomas.

## Competing interests

No potential conflict of interest relevant to this article is reported by any of the authors.

## Authors’ contributions

VL participated in acquiring clinical and laboratory data, data analysis and interpretation, and drafted the manuscript. PG-P, JS and JCC participated in acquiring laboratory data, data analysis and data interpretation and drafted the manuscript. AS, FA, AV, JB, and OH acquired urinary and tissue samples and their follow-up clinical information. MSC participated in study design and coordination, data analysis and interpretation and final writing of the manuscript. All authors read and approved the final manuscript.

## Supplementary Material

Additional file 1: Figure S1 Experimental design. Array-CGH. A. Urinary DNAs were subjected to array-CGH to identify genomic copy number differences between bladder cancer patients (n = 14) and control individuals (n = 8). Validation analyses. Two different approaches were applied to evaluate the association of a selected candidate gene mapping at such genomic imbalances with tumor progression and other clinicopathologic variables. B. FISH analyses were optimized to validate the copy number gain of the candidate gene PFND2 on paraffin embedded tumors paired to the urinary specimens (a representative case is shown) spotted on tissue arrays that also contained independent sets of bladder tumors (n = 181). C. In addition, IHC analyses were carried out on the paired tumors of the urinary specimens under analyses (a representative case is shown), and on the above mentioned tissue arrays. These FISH and IHC analyses served to validate associations of PFND2 with clinicopathologic variables. D. Western blot analyses were performed using protein extracts from nine bladder cancer cell lines derived from TCCs of the bladder of early stage (RT4), low grade (5637), invasive (T24, J82, UM-UC-3, RT112, EJ138), metastatic (TCC-SUP), and squamous cell carcinoma (ScaBER), to confirm the specificity of the PFND2 antibody utilized in the study. The antibody was accepted because of displaying a single predominant band at the expected molecular weight (16 KDa). Moreover, invasive and metastatic cell lines derived from advanced bladder tumors showed higher PFND2 expression than those derived from early stage and low grade tumors. Click here for file

Additional file 2: Figure S2 Evaluation of the reproducibility of array-CGH on urinary DNA. A. Summary ideogram given by the CGH Analytics software for one representative example comparing array-CGH results using 250 ng *versus* 500 ng as initial amount of DNA. Average log_2_ ratio values along the chromosomes are represented by the red (250 ng) and blue (500 ng) lines. Displacement of the tracing of these red and blue lines to the right or left represents genomic gains or losses, respectively, which display in parallel. B. Genomic profile given by the InSilicoArray CGH software comparing the hybridizaton of two independent reference urinary pools labelled against each other. The two urinary pools consisted of eight samples from healthy donors and individuals with no evidence of disease. Average log_10_ ratio values of the CNVs along the chromosome are represented by the blue line. Displacement of the tracing of this blue line to the right or left represents genomic gains or losses, respectively. Lack of displacement represents similar genomic profiles of the reverse labeled pools. The profiles are ordered from chromosome 1 to 22, including chromosomes X and Y as well. Click here for file

Additional file 3: Figure S3 Urinary genomic DNA profiles obtained by array-CGH for all the urinary specimens of the bladder cancer cases under analyses ordered by tumor staging and presented as individual ideograms given by the CGH Analytics software. Moving average log_2_ ratio values along the chromosome are represented by the red line. Displacement of the tracing of this red line to the right or left represents genomic gains or losses, respectively. The ideograms are ordered from chromosome 1 to 22, including chromosomes X and Y. Click here for file

Additional file 4: Table S1 Complete set of known genes mapping to the minimally recurrent regions. Click here for file

## References

[B1] Siegel R Naishadham D Jemal A Cancer statistics 2012 CA Cancer J Clin 2012 62 10 29 10.3322/caac.20138 22237781

[B2] Kirkali Z Chan T Manoharan M Algaba F Busch C Cheng L Kiemeney L Kriegmair M Montironi R Murphy WM Sesterhenn IA Tachibana M Weider J Bladder cancer: epidemiology, staging and grading, and diagnosis Urology 2005 66 4 34 16399414 10.1016/j.urology.2005.07.062

[B3] Kallioniemi A Kallioniemi OP Sudar D Rutovitz D Gray JW Waldman F Pinkel D Comparative genomic hybridization for molecular cytogenetic analysis of solid tumors Science 1992 258 818 821 10.1126/science.1359641 1359641

[B4] Pinkel D Segraves R Sudar D Clark S Poole I Kowbel D Collins C Kuo WL Chen C Zhai Y Dairkee SH Ljung BM Gray JW Albertson DG High resolution analysis of DNA copy number variation using comparative genomic hybridization to microarrays Nat Genet 1998 20 207 211 10.1038/2524 9771718

[B5] Kallioniemi A Kallioniemi OP Citro G Sauter G DeVries S Kerschmann R Caroll P Waldman F Identification of gains and losses of DNA sequences in primary bladder cancer by comparative genomic hybridization Genes Chromosomes Cancer 1995 12 213 219 10.1002/gcc.2870120309 7536461

[B6] Voorter C Joos S Bringuier PP Vallinga M Poddighe P Schalken J du Manoir S Ramaekers F Lichter P Hopman A Detection of chromosomal imbalances in transitional cell carcinoma of the bladder by comparative genomic hybridization Am J Pathol 1995 146 1341 1354 7778674 PMC1870895

[B7] Obermann EC Junker K Stoehr R Dietmaier W Zaak D Schubert J Hofstaedter F Knuechel R Hartmann A Frequent genetic alterations in flat urothelial hyperplasias and concomitant papillary bladder cancer as detected by CGH, LOH, and FISH analyses J Pathol 2003 199 50 57 10.1002/path.1259 12474226

[B8] Constantinou M Binka-Kowalska A Borkowska E Zajac E Jałmuzna P Matych J Nawrocka A Kałuzewski B Application of multiplex FISH, CGH and MSSCP techniques for cytogenetic and molecular analysis of transitional cell carcinoma (TCC) cells in voided urine specimens J Appl Genet 2006 47 273 275 10.1007/BF03194636 16877809

[B9] Junker K van Oers JM Zwarthoff EC Kania I Schubert J Hartmann A Fibroblast growth factor receptor 3 mutations in bladder tumors correlate with low frequency of chromosome alterations Neoplasia 2008 10 1 7 10.1593/neo.07178 18231634 PMC2213896

[B10] El-Rifai W Kamel D Larramendy ML Shoman S Gad Y Baithun S El-Awady M Eissa S Khaled H Soloneski S Sheaff M Knuutila S DNA copy number changes in Schistosoma-associated and non-Schistosoma-associated bladder cancer Am J Pathol 2000 156 871 878 10.1016/S0002-9440(10)64956-5 10702404 PMC1876852

[B11] Richter J Jiang F Görög JP Sartorius G Egenter C Gasser TC Moch H Mihatsch MJ Sauter G Marked genetic differences between stage pTa and stage pT1 papillary bladder cancer detected by comparative genomic hybridization Cancer Res 1997 57 2860 2864 9230190

[B12] Richter J Beffa L Wagner U Schraml P Gasser TC Moch H Mihatsch MJ Sauter G Patterns of chromosomal imbalances in advanced urinary bladder cancer detected by comparative genomic hybridization Am J Pathol 1998 153 1615 1621 10.1016/S0002-9440(10)65750-1 9811354 PMC1853402

[B13] Simon R Bürger H Brinkschmidt C Böcker W Hertle L Terpe HJ Chromosomal aberrations associated with invasion in papillary superficial bladder cancer J Pathol 1998 185 345 351 10.1002/(SICI)1096-9896(199808)185:4<345::AID-PATH109>3.0.CO;2-0 9828832

[B14] Muscheck M Abol-Enein H Chew K Moore D 2nd Bhargava V Ghoneim MA Carroll PR Waldman FM Comparison of genetic changes in schistosome-related transitional and squamous bladder cancers using comparative genomic hybridization Carcinogenesis 2000 21 1721 1726 10.1093/carcin/21.9.1721 10964104

[B15] Fadl-Elmula I Kytola S Leithy ME Abdel-Hameed M Mandahl N Elagib A Ibrahim M Larsson C Heim S Chromosomal aberrations in benign and malignant bilharzia-associated bladder lesions analyzed by comparative genomic hybridization BMC Cancer 2002 2 5 10.1186/1471-2407-2-5 11914143 PMC101388

[B16] Harding M Arden K Gildea JW Gildea JJ Perlman EJ Viars C Theodorescu D Functional Genomic comparison of lineage-related human bladder cancer cell lines with differing tumorigenic and metastatic potentials by spectral karyotyping, comparative genomic hybridization, and a novel method of positional expression profiling Cancer Res 2002 62 6981 6989 12460916

[B17] Wang X Ling MT Guan XY Tsao SW Cheung HW Lee DT Wong YC Identification of a novel function of TWIST, a bHLH protein, in the development of acquired taxol resistance in human cancer cells Oncogene 2004 23 474 482 10.1038/sj.onc.1207128 14724576

[B18] Wu Z Siadaty MS Riddick G Frierson HF Jr Lee JK Golden W Knuutila S Hampton GM El-Rifai W Theodorescu D A novel method for gene expression mapping of metastatic competence in human bladder cancer Neoplasia 2006 8 181 189 10.1593/neo.05727 16611411 PMC1578518

[B19] Veltman JA Fridlyand J Pejavar S Olshen AB Korkola JE DeVries S Carroll P Kuo WL Pinkel D Albertson D Cordon-Cardo C Jain AN Waldman FM Array-based comparative genomic hybridization for genome-wide screening of DNA copy number in bladder tumors Cancer Res 2003 63 2872 2880 12782593

[B20] Hupé P Stransky N Thiery JP Radvanyi F Barillot E Analysis of array CGH data: from signal ratio to gain and loss of DNA regions Bioinformatics 2004 20 3413 3422 10.1093/bioinformatics/bth418 15381628

[B21] Blaveri E Brewer JL Roydasgupta R Fridlyand J DeVries S Koppie T Pejavar S Mehta K Carroll P Simko JP Waldman FM Bladder cancer stage and outcome by array-based comparative genomic hybridization Clin Cancer Res 2005 11 7012 7022 10.1158/1078-0432.CCR-05-0177 16203795

[B22] Stransky N Vallot C Reyal F Bernard-Pierrot I de Medina SG Segraves R de Rycke Y Elvin P Cassidy A Spraggon C Graham A Southgate J Asselain B Allory Y Abbou CC Albertson DG Thiery JP Chopin DK Pinkel D Radvanyi F Regional copy number-independent deregulation of transcription in cancer Nat Genet 2006 38 1386 1396 10.1038/ng1923 17099711

[B23] Yamamoto Y Chochi Y Matsuyama H Eguchi S Kawauchi S Furuya T Oga A Kang JJ Naito K Sasaki K Gain of 5p15.33 is associated with progression of bladder cancer Oncology 2007 72 132 138 18025801 10.1159/000111132

[B24] Hurst CD Tomlinson DC Williams SV Platt FM Knowles MA Inactivation of the Rb pathway and overexpression of both isoforms of E2F3 are obligate events in bladder tumours with 6p22 amplification Oncogene 2008 27 2716 2727 10.1038/sj.onc.1210934 18037967 PMC2387074

[B25] Hurst CD Fiegler H Carr P Williams S Carter NP Knowles MA High-resolution analysis of genomic copy number alterations in bladder cancer by microarray-based comparative genomic hybridization Oncogene 2004 23 2250 2263 10.1038/sj.onc.1207260 14968109

[B26] Shinoda Y Kozaki K Imoto I Obara W Tsuda H Mizutani Y Shuin T Fujioka T Miki T Inazawa J Association of KLK5 overexpression with invasiveness of urinary bladder carcinoma cells Cancer Sci 2007 98 1078 1086 10.1111/j.1349-7006.2007.00495.x 17459052 PMC11158320

[B27] Vauhkonen H Böhling T Eissa S Shoman S Knuutila S Can bladder adenocarcinomas be distinguished from schistosomiasis-associated bladder cancers by using array comparative genomic hybridization analysis? Cancer Genet Cytogenet 2007 177 153 157 10.1016/j.cancergencyto.2007.06.017 17854674

[B28] Larré S Camparo P Comperat E Gil Diez De Medina S Traxer O Roupret M Sebe P Cancel-Tassin G Sighar K Lozach F Cussenot O Diagnostic, staging, and grading of urothelial carcinomas from urine: performance of BCA-1, a mini-array comparative genomic hybridisation-based test Eur Urol 2011 59 250 257 10.1016/j.eururo.2010.10.007 21056532

[B29] Herrero J Al-Shahrour F Díaz-Uriarte R Mateos A Vaquerizas JM Santoyo J Dopazo J A web-based resource for microarray gene expression data analysis Nucleic Acids Res 2003 31 3461 3467 10.1093/nar/gkg591 12824345 PMC168997

[B30] Benjamini Y Hochberg Y Controlling the false discovery rate: a practical and powerful approach to multiple testing J Royal Statistical Society 1995 57 289 300

[B31] Sanchez-Carbayo M Socci ND Charytonowicz E Lu M Prystowsky M Childs G Cordon-Cardo C Molecular profiling of bladder cancer using cDNA microarrays: defining histogenesis and biological phenotypes Cancer Res 2002 62 6973 6980 12460915

[B32] Dawson-Saunders B Trapp RG Basic and Clinical Biostatistics 1994 2 Norwalk, Connecticut: Appleton & Lange

[B33] Dalbagni G Presti J Reuter V Fair WR Cordon-Cardo C Genetic alterations in bladder cancer Lancet 1993 324 469 471 8102431 10.1016/0140-6736(93)91595-d

[B34] Savelieva E Belair CD Newton MA DeVries S Gray JW Waldman F Reznikoff CA 20q gain associates with immortalization: 20q13.2 amplification correlates with genome instability in human papillomavirus 16 E7 transformed human uroepithelial cells Oncogene 1997 14 551 560 10.1038/sj.onc.1200868 9053853

[B35] Richter J Wagner U Kononen J Fijan A Bruderer J Schmid U Ackermann D Maurer R Alund G Knönagel H Rist M Wilber K Anabitarte M Hering F Hardmeier T Schönenberger A Flury R Jäger P Fehr JL Schraml P Moch H Mihatsch MJ Gasser T Kallioniemi OP Sauter G High-throughput tissue microarray analysis of cyclin E gene amplification and overexpression in urinary bladder cancer Am J Pathol 2000 157 787 794 10.1016/S0002-9440(10)64592-0 10980118 PMC1885698

[B36] Bruch J Wöhr G Hautmann R Mattfeldt T Brüderlein S Möller P Sauter S Hameister H Vogel W Paiss T Chromosomal changes during progression of transitional cell carcinoma of the bladder and delineation of the amplified interval on chromosome arm 8q Genes Chromosomes Cancer 1998 23 167 174 10.1002/(SICI)1098-2264(199810)23:2<167::AID-GCC10>3.0.CO;2-L 9739020

[B37] Yeager TR De Vries S Jarrard DF Kao C Nakada SY Moon TD Bruskewitz R Stadler WM Meisner LF Gilchrist KW Newton MA Waldman FM Reznikoff CA Overcoming cellular senescence in human cancer pathogenesis Genes Dev 1998 12 163 174 10.1101/gad.12.2.163 9436977 PMC316442

[B38] Koo SH Kwon KC Ihm CH Jeon YM Park JW Sul CK Detection of genetic alterations in bladder tumors by comparative genomic hybridization and cytogenetic analysis Cancer Genet Cytogenet 1999 110 87 93 10.1016/S0165-4608(98)00193-9 10214355

[B39] Terracciano L Richter J Tornillo L Beffa L Diener PA Maurer R Gasser TC Moch H Mihatsch MJ Sauter G Chromosomal imbalances in small cell carcinomas of the urinary bladder J Pathol 1999 189 230 235 10.1002/(SICI)1096-9896(199910)189:2<230::AID-PATH407>3.0.CO;2-8 10547580

[B40] Williams SV Adams J Coulter J Summersgill BM Shipley J Knowles MA Assessment by M-FISH of karyotypic complexity and cytogenetic evolution in bladder cancer *in vitro* Genes Chromosomes Cancer 2005 43 315 328 10.1002/gcc.20166 15846775

[B41] Appanna TC Doak SH Jenkins SA Kynaston HG Stephenson TP Parry JM Comparative genomic hybridization (CGH) of augmentation cystoplasties Int J Urol 2007 14 539 544 10.1111/j.1442-2042.2006.01724.x 17593101

[B42] Dahse R Gärtner D Werner W Schubert J Junker K P53 mutations as an identification marker for the clonal origin of bladder tumors and its recurrences Oncol Rep 2003 10 2033 2037 14534739

[B43] Pinkel D Albertson DG Array comparative genomic hybridization and its applications in cancer Nat Genet 2005 37 S11 S17 10.1038/ng1569 15920524

[B44] Vainberg IE Lewis SA Rommelaere H Ampe C Vandekerckhove J Klein HL Cowan NJ Prefoldin, a chaperone that delivers unfolded proteins to cytosolic chaperonin Cell 1998 93 863 873 10.1016/S0092-8674(00)81446-4 9630229

[B45] Hansen WJ Cowan NJ Welch WJ Prefoldin-nascent chain complexes in the folding of cytoskeletal proteins J Cell Biol 1999 145 265 277 10.1083/jcb.145.2.265 10209023 PMC2133115

[B46] Muhua L Karpova TS Cooper JA A yeast actin-related protein homologous to that in vertebrate dynactin complex is important for spindle orientation and nuclear migration Cell 1994 78 669 679 10.1016/0092-8674(94)90531-2 8069915

[B47] Moreau V Madania A Martin RP Winson B The Saccharomyces cerevisiae actin-related protein Arp2 is involved in the actin cytoskeleton J Cell Biol 1996 134 117 132 10.1083/jcb.134.1.117 8698808 PMC2120930

[B48] Fryer CJ Archer TK Chromatin remodelling by the glucocorticoid receptor requires the BRG1 complex Nature 1998 393 88 91 10.1038/30032 9590696

[B49] Peterson CL Zhao Y Chait BT Subunits of the yeast SWI/SNF complex are members of the actin-related protein (ARP) family J Biol Chem 1998 273 23641 23644 10.1074/jbc.273.37.23641 9726966

[B50] Takahashi M Watari E Shinya E Shimizu T Takahashi H Suppression of virus replication via down-modulation of mitochondrial short chain enoyl-CoA hydratase in human glioblastoma cells Antiviral Res 2007 75 152 158 10.1016/j.antiviral.2007.02.002 17395278

[B51] Collins C Volik S Kowbel D Ginzinger D Ylstra B Cloutier T Hawkins T Predki P Martin C Wernick M Kuo WL Alberts A Gray JW Comprehensive genome sequence analysis of a breast cancer amplicon Genome Res 2001 11 1034 1042 10.1101/gr.GR1743R 11381030 PMC311107

[B52] Alldinger I Dittert D Peiper M Fusco A Chiappetta G Staub E Lohr M Jesnowski R Baretton G Ockert D Saeger HD Grützmann R Pilarsky C Gene expression analysis of pancreatic cell lines reveals genes overexpressed in pancreatic cancer Pancreatology 2005 5 370 379 10.1159/000086537 15983444

[B53] Ostrov DA Barnes CL Smith LE Binns S Brusko TM Brown AC Quint PS Litherland SA Roopenian DC Iczkowski KA Characterization of HKE2: an ancient antigen encoded in the major histocompatibility complex Tissue Antigens 2007 69 181 188 10.1111/j.1399-0039.2006.00730.x 17257322

[B54] Kurata M Maesako Y Ueda C Nishikori M Akasaka T Uchiyama T Ohno H Characterization of t(3;6)(q27;p21) breakpoints in B-cell non-Hodgkin’s lymphoma and construction of the histone H4/BCL6 fusion gene, leading to altered expression of Bcl-6 Cancer Res 2002 62 6224 6230 12414651

[B55] Myung JK Afjehi-Sadat L Felizardo-Cabatic M Slavc I Lubec G Expressional patterns of chaperones in ten human tumor cell lines Proteome Sci 2004 2 8 10.1186/1477-5956-2-8 15598346 PMC543454

[B56] Cimmino F Spano D Capasso M Zambrano N Russo R Zollo M Iolascon A Comparative proteomic expression profile in all-trans retinoic acid differentiated neuroblastoma cell line J Proteome Res 2007 6 2550 2564 10.1021/pr060701g 17559250

