# Supplementary material for: Identification of prefoldin amplification (1q23.3-q24.1) in bladder cancer using comparative genomic hybridization (CGH) arrays of urinary DNA
Source: J Transl Med. 2013 Aug 1;11:182. doi: 10.1186/1479-5876-11-182 (PMC3750577; doi:10.1186/1479-5876-11-182)
Supplement: Additional file 1: Figure S1 — Experimental design. Array-CGH. A. Urinary DNAs were subjected to array-CGH to identify genomic copy number differences between bladder cancer patients (n = 14) and control individuals (n = 8). Validation analyses. Two different approaches were applied to evaluate the association of a selected candidate gene mapping at such genomic imbalances with tumor progression and other clinicopathologic variables. B. FISH analyses were optimized to validate the copy number gain of the candidate gene PFND2 on paraffin embedded tumors paired to the urinary specimens (a representative case is shown) spotted on tissue arrays that also contained independent sets of bladder tumors (n = 181). C. In addition, IHC analyses were carried out on the paired tumors of the urinary specimens under analyses (a representative case is shown), and on the above mentioned tissue arrays. These FISH and IHC analyses served to validate associations of PFND2 with clinicopathologic variables. D. Western blot analyses were performed using protein extracts from nine bladder cancer cell lines derived from TCCs of the bladder of early stage (RT4), low grade (5637), invasive (T24, J82, UM-UC-3, RT112, EJ138), metastatic (TCC-SUP), and squamous cell carcinoma (ScaBER), to confirm the specificity of the PFND2 antibody utilized in the study. The antibody was accepted because of displaying a single predominant band at the expected molecular weight (16 KDa). Moreover, invasive and metastatic cell lines derived from advanced bladder tumors showed higher PFND2 expression than those derived from early stage and low grade tumors. [file 1479-5876-11-182-S1.ppt]

## Slide 1
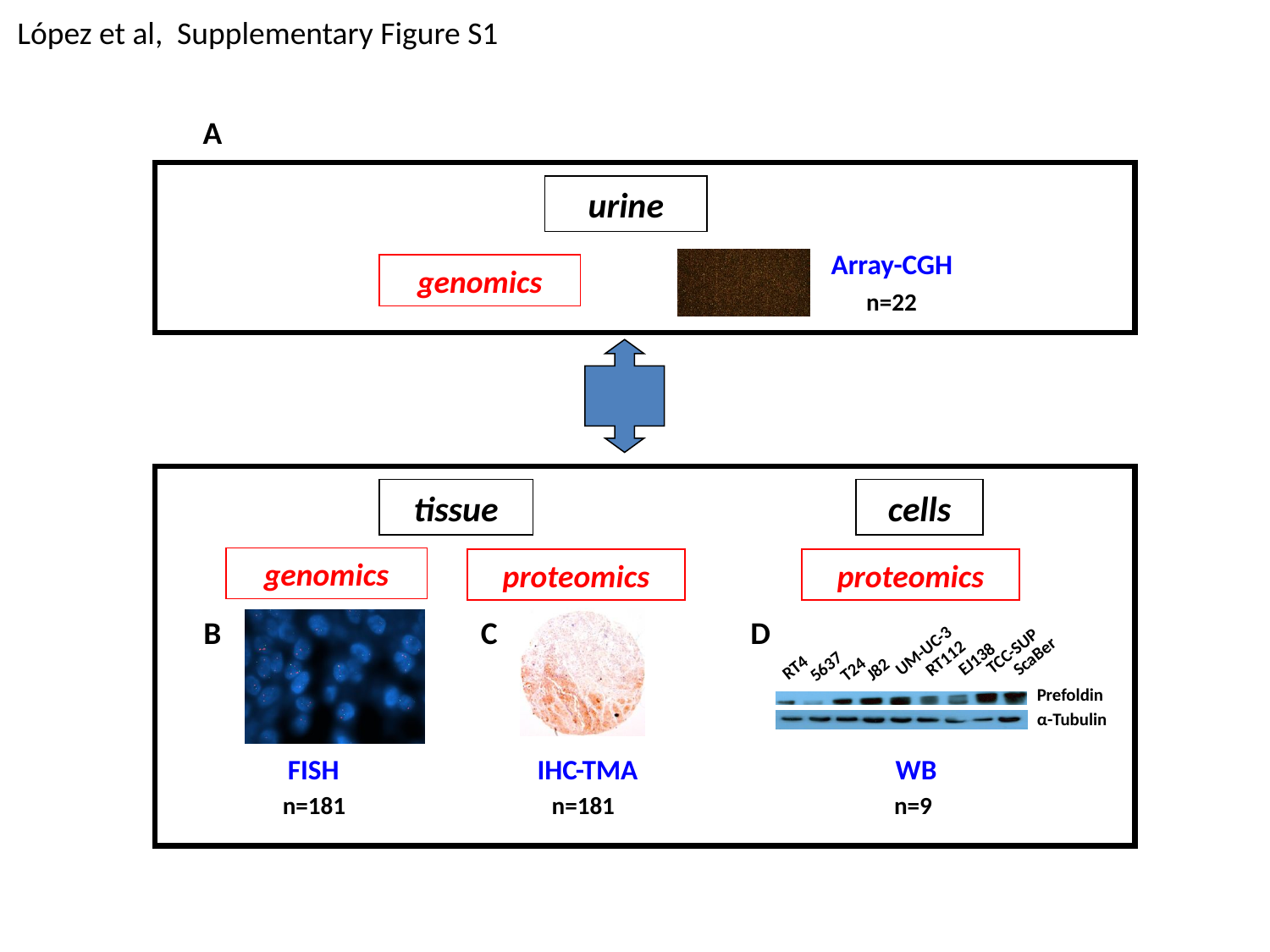

López et al, Supplementary Figure S1
A
urine
Array-CGH
genomics
n=22
tissue
cells
genomics
proteomics
proteomics
B
C
D
UM-UC-3
TCC-SUP
ScaBer
EJ138
5637
RT4
J82
RT112
T24
Prefoldin
α-Tubulin
WB
FISH
IHC-TMA
n=181
n=181
n=9
