# Supplementary figures and images for: Identification of prefoldin amplification (1q23.3-q24.1) in bladder cancer using comparative genomic hybridization (CGH) arrays of urinary DNA
Source: J Transl Med. 2013 Aug 1;11:182. doi: 10.1186/1479-5876-11-182 (PMC3750577; doi:10.1186/1479-5876-11-182)

## Slide 1
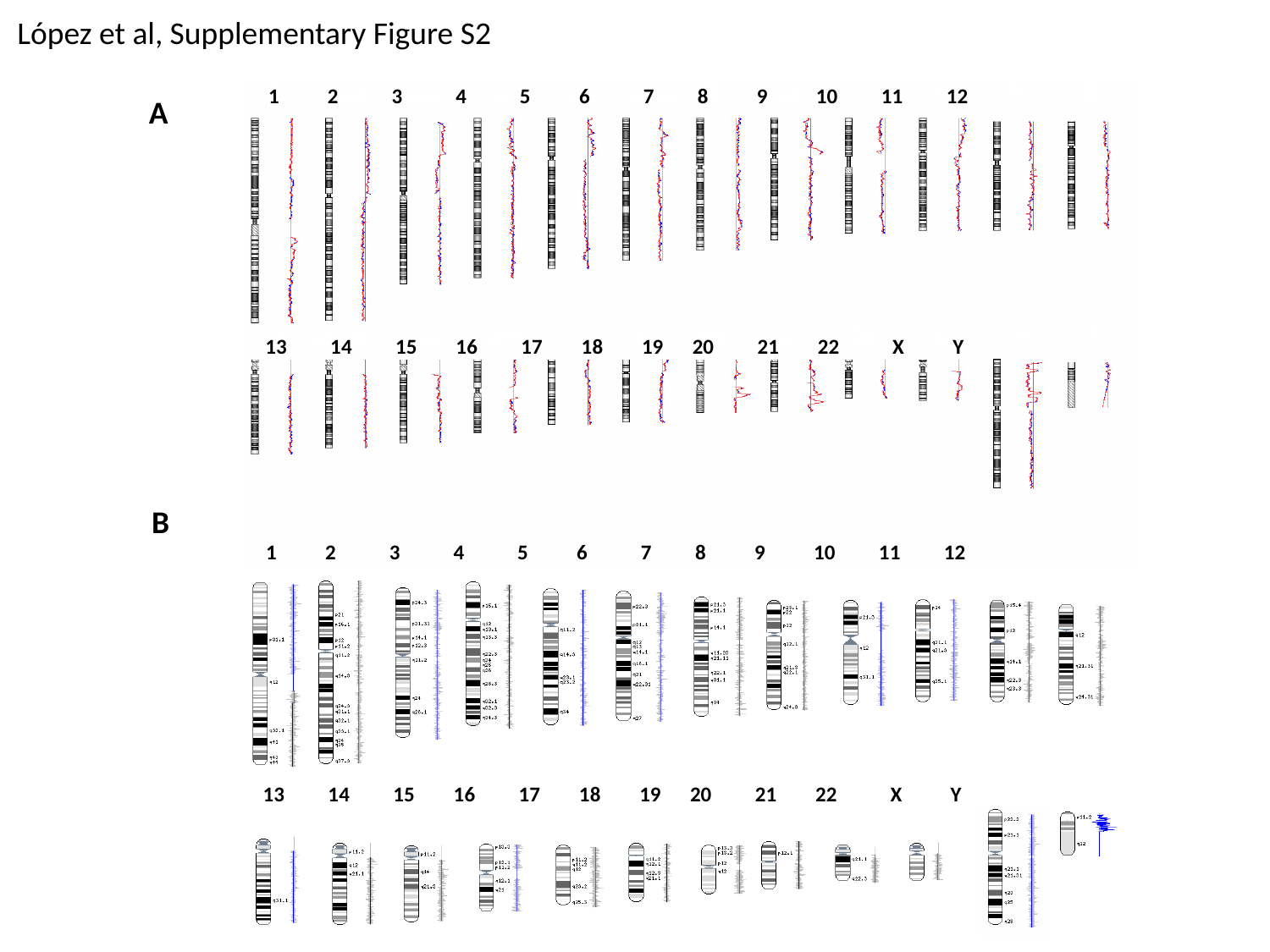

López et al, Supplementary Figure S2
 1 2 3 4 5 6 7 8 9 10 11 12
 13 14 15 16 17 18 19 20 21 22 X Y
A
B
 1 2 3 4 5 6 7 8 9 10 11 12
 13 14 15 16 17 18 19 20 21 22 X Y

Supplement: Additional file 2: Figure S2 — Evaluation of the reproducibility of array-CGH on urinary DNA. A. Summary ideogram given by the CGH Analytics software for one representative example comparing array-CGH results using 250 ng versus 500 ng as initial amount of DNA. Average log2 ratio values along the chromosomes are represented by the red (250 ng) and blue (500 ng) lines. Displacement of the tracing of these red and blue lines to the right or left represents genomic gains or losses, respectively, which display in parallel. B. Genomic profile given by the InSilicoArray CGH software comparing the hybridizaton of two independent reference urinary pools labelled against each other. The two urinary pools consisted of eight samples from healthy donors and individuals with no evidence of disease. Average log10 ratio values of the CNVs along the chromosome are represented by the blue line. Displacement of the tracing of this blue line to the right or left represents genomic gains or losses, respectively. Lack of displacement represents similar genomic profiles of the reverse labeled pools. The profiles are ordered from chromosome 1 to 22, including chromosomes X and Y as well. [file 1479-5876-11-182-S2.ppt]
