# Supplementary material for: Identification of prefoldin amplification (1q23.3-q24.1) in bladder cancer using comparative genomic hybridization (CGH) arrays of urinary DNA
Source: J Transl Med. 2013 Aug 1;11:182. doi: 10.1186/1479-5876-11-182 (PMC3750577; doi:10.1186/1479-5876-11-182)
Supplement: Additional file 3: Figure S3 — Urinary genomic DNA profiles obtained by array-CGH for all the urinary specimens of the bladder cancer cases under analyses ordered by tumor staging and presented as individual ideograms given by the CGH Analytics software. Moving average log2 ratio values along the chromosome are represented by the red line. Displacement of the tracing of this red line to the right or left represents genomic gains or losses, respectively. The ideograms are ordered from chromosome 1 to 22, including chromosomes X and Y. [file 1479-5876-11-182-S3.ppt]

## Slide 1
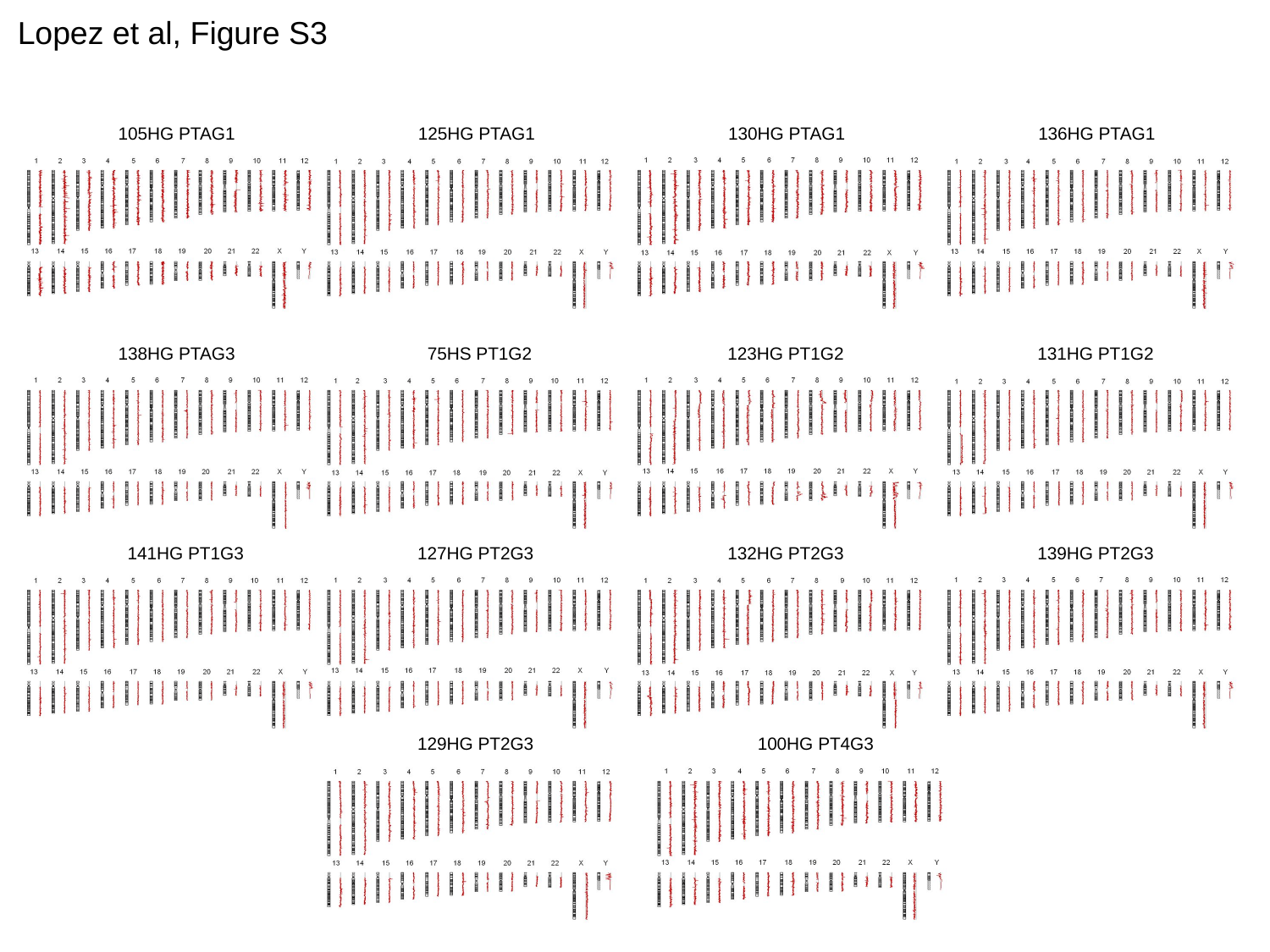

Lopez et al, Figure S3
105HG PTAG1
125HG PTAG1
130HG PTAG1
136HG PTAG1
138HG PTAG3
75HS PT1G2
123HG PT1G2
131HG PT1G2
141HG PT1G3
127HG PT2G3
132HG PT2G3
139HG PT2G3
129HG PT2G3
100HG PT4G3
